# 2285: Analysis of racial disparity in the whole blood and plasma of healthy volunteers using rotational thromboelastometry

**DOI:** 10.1017/cts.2017.126

**Published:** 2018-05-10

**Authors:** Maissaa Janbain, Anita Madison, Cindy Leissinger

## Abstract

OBJECTIVES/SPECIFIC AIMS: To explore the racial differences in rotational thromboelastometry findings using whole blood and plasma samples from healthy volunteers. METHODS/STUDY POPULATION: We studied a cohort of patients at Tulane University Hospitals who came into the pre-op clinic to get blood drawn for labs. The cohort included a total of 44 patients who were otherwise healthy adult volunteers with no history of cardiovascular nor thromboembolic events, 30 African Americans and 14 Caucasians. Patients who required lab work for their upcoming surgery were asked to participate in the study by giving a sample of blood collecting in a light blue-top sodium citrate tube. We excluded patients who were currently on any anticoagulation or antiplatelet medications. We also excluded those with current or previous history of cancer, those with known bleeding disorder, and those who were on chronic transfusion protocol, or had received a blood transfusion within the last 21 days. Data collection was carried out after informed consent was obtained; we collected citrated whole-blood (WB) samples. WB samples were processed within 3 hours of phlebotomy. Platelet free plasma, obtained after centrifugation at 2500 cGy of whole blood for 20 minutes, was kept frozen at −70°C. Frozen plasma was thawed at 37°C for 5 minutes before testing. Samples were recalcified with star-tem reagent, and then the in-tem reagent was added. The latter contains an optimized concentration of ellagic acid and partial thromboplastin phospholipid from rabbit brain. Thromboelastometry (ROTEM) parameters including clotting time, clot formation time, alpha angle, maximum clot firmness, and Lysis Index after 30 and 45 minutes were determined. Data was then retrieved from the ROTEM database and put into an Excel sheet to be analyzed. RESULTS/ANTICIPATED RESULTS: Our results showed that the CFT was higher in both the plasma and the WB of Caucasians when compared with African Americans with a difference between means 137.5±233.7 (*p*=0.56) and 11±7.85 (*p*=0.168), respectively; while MCF was increased in the WB and plasma of AA with a difference between means of 1.719±1.974 (*p*=0.38) and 5.37±2.49 (*p*=0.037), respectively. In other words, the plasma of Caucasians did seem to take longer to reach the maximum firmness (however not statistically significant *p*>0.05), while the maximum clot firmness was significantly higher in plasma of AA. In summary and compatibly with the previously published data, our results showed significantly increased prothrombotic profile in the plasma of African Americans when compared with Caucasians. DISCUSSION/SIGNIFICANCE OF IMPACT: This reinforces the role of the whole vascular system and the interaction between its different components in the pathophysiology of thromboembolic events. In one case control study, African ethnicity was associated with increased risk of DVT in parallel with significantly increased peak thrombin on thrombin generation when compared with Caucasians. With our preliminary results, we confirm these data using another tool for the assessment of the plasma in addition to comparing WB samples too. More prospective studies, with higher number of subjects evaluating the value of the results in predicting the risk of development of thromboembolic events in different ethnicities, are needed for better understanding of this disease. In addition, thromboelastometry might require adjustment for ethnicity in studies evaluating ethnically diverse populations.

